# Postural Ataxia in Cerebellar Downbeat Nystagmus: Its Relation to Visual, Proprioceptive and Vestibular Signals and Cerebellar Atrophy

**DOI:** 10.1371/journal.pone.0168808

**Published:** 2017-01-05

**Authors:** Christoph Helmchen, Jan-Birger Kirchhoff, Martin Göttlich, Andreas Sprenger

**Affiliations:** 1 Department of Neurology, University of Lübeck, Lubeck, Germany; 2 Institute of Psychology II, University of Lübeck, Lubeck, Germany; Tokai University, JAPAN

## Abstract

**Background:**

The cerebellum integrates proprioceptive, vestibular and visual signals for postural control. Cerebellar patients with downbeat nystagmus (DBN) complain of unsteadiness of stance and gait as well as blurred vision and oscillopsia.

**Objectives:**

The aim of this study was to elucidate the differential role of visual input, gaze eccentricity, vestibular and proprioceptive input on the postural stability in a large cohort of cerebellar patients with DBN, in comparison to healthy age-matched control subjects.

**Methods:**

Oculomotor (nystagmus, smooth pursuit eye movements) and postural (postural sway speed) parameters were recorded and related to each other and volumetric changes of the cerebellum (voxel-based morphometry, SPM).

**Results:**

Twenty-seven patients showed larger postural instability in all experimental conditions. Postural sway increased with nystagmus in the eyes closed condition but not with the eyes open. Romberg’s ratio remained stable and was not different from healthy controls. Postural sway did not change with gaze position or graviceptive input. It increased with attenuated proprioceptive input and on tandem stance in both groups but Romberg’s ratio also did not differ. Cerebellar atrophy (vermal lobule VI, VIII) correlated with the severity of impaired smooth pursuit eye movements of DBN patients.

**Conclusions:**

Postural ataxia of cerebellar patients with DBN cannot be explained by impaired visual feedback. Despite oscillopsia visual feedback control on cerebellar postural control seems to be preserved as postural sway was strongest on visual deprivation. The increase in postural ataxia is neither related to modulations of single components characterizing nystagmus nor to deprivation of single sensory (visual, proprioceptive) inputs usually stabilizing stance. Re-weighting of multisensory signals and/or inappropriate cerebellar motor commands might account for this postural ataxia.

## Introduction

Downbeat nystagmus (DBN) is the most common form of acquired persisting spontaneous nystagmus. Patients usually complain of unsteadiness of gait and stance as well as blurred vision and oscillopsia [1]. DBN is a typical ocular motor sign in patients with cerebellar (i.e. flocculus) impairment. It consists of an upward drift of the eye that does not depend on vertical head position (spontaneous drift), a gravity-dependent component [1], and a gaze-evoked drift [2, 3]. It is commonly believed that motion of the retina (retinal slip) affects postural stability. As nystagmus and oscillopsia in DBN usually increase with eccentric gaze one might suspect increase in postural imbalance and gait ataxia [4]. Moreover, eccentric eye positions in healthy subjects elicit eye deviation from the intended path [5]. In turn, nystagmus suppression, at least in vestibular disease, may reduce postural sway [6].

Up to now, however, it remained unclear whether postural ataxia in DBN is related to the (i) severity of visual impairment by oscillopsia, (ii) to nystagmus amplitude irrespective of retinal slip or (iii) to disease severity reflected by deficient cerebellar control on stance, irrespective of visual feedback.

Moreover, gait disturbances in DBN have been found not to be purely related to impaired visual control since walking in darkness (eyes closed) was significantly worse than in age-matched controls [7].

The aim of this study was therefore to elucidate the differential role of gaze eccentricity, gravity (head position dependency) and proprioceptive input on the postural stability of idiopathic DBN patients in comparison to healthy age-matched control subjects and its relation to morphological changes of the cerebellum. If DBN was related to the severity of visual impairment by oscillopsia, postural instability should increase stronger with the eyes open than with the eyes closed (*Hypothesis #1*). If postural ataxia was independent of oscillopsia (*Hypothesis #2*) there should be either no context-dependent (eyes open vs. closed) difference or a larger postural sway in the dark as compared to age-matched healthy controls. This would reflect either an influence of eye movement signals on postural stability in the absence of visual feedback, e.g. efference copy signals [8], or a motor mechanism independent from retinal slip and eye movement signals.

## Methods

### Participants

Thirty-one DBN patients were enrolled in this study and compared with 27 healthy control subjects. Mean disease duration was 5.7±3.0 (SD) years. Participants were recruited from 2013 to 2014. They gave their written informed consent to participate in this study which was approved by The University of Luebeck Ethics Committee (#12–154). Clinical data of patients are listed in S1 Table. We excluded 4 DBN patients with clinical evidence for polyneuropathy or uni / bilateral vestibulopathy. Thus, the remaining 27 DBN patients (mean age: 73.7±8.7 (SD) years) had no (additional) vestibular and proprioceptive deficit and were classified as idiopathic since no causative factors could be determined [9]. Vestibular testing in patients included the head impulse test of the horizontal vestibulo-ocular reflex which revealed normal vestibular functions in all participants. All patients underwent at least one cranial MRI. None of them showed focal structural abnormalities that could account for the cerebellar syndrome. Some of the patients received various medications over the last years which were discontinued before this study to exclude drug-related side effects: potassium channel-blockers, i.e. 3,4-diaminopyridine (3 x 5 mg/day), 4-aminopyridine 3 x 5 mg/day or 2 x 10 mg/day of its slow release form fampridine; chlorzoxazone 3 x 500 mg, or acetyl-DL-leucine (3 g/day). No patient received any “ataxia-modulating drug” during the study.

Besides neurological, neuro-otological and neuro-ophthalmological examination patients were tested by using different clinical scores of cerebellar impairment, i.e. scale for the assessment and rating of ataxia (SARA) and the Tinetti Assessment Test [10, 11], vestibular, i.e. vertigo handicap questionnaire (VHQ)[12] and Vertigo Symptom Scale (VSS) [13], and cognitive function (Montreal Cognitive Assessment, MOCA) (S2 Table).

Twenty-seven age-matched healthy subjects [mean age: 70.3±4.7 (SD) years, no significant difference to patients] with no detectable disease affecting postural control served as controls. Oculomotor behavior, i.e. gaze stability, saccades and smooth pursuit eye movements, was recorded in both groups by a video-based eye tracker (EyeLink II, SR Research, ON, CA) and analysed [14, 15]. A laser target was used as stimulus (size 0.1°) which was projected on a screen 140 cm in front of the participants. DBN was tested and analyzed for eccentric horizontal (± 20°) and vertical (±12°) target positions [2]. Smooth pursuit eye movements were recorded using a target that moved sinusoidally in horizontal and vertical direction (0.2 Hz, ±16° amplitude).

### Experimental conditions

**Fig 1:**
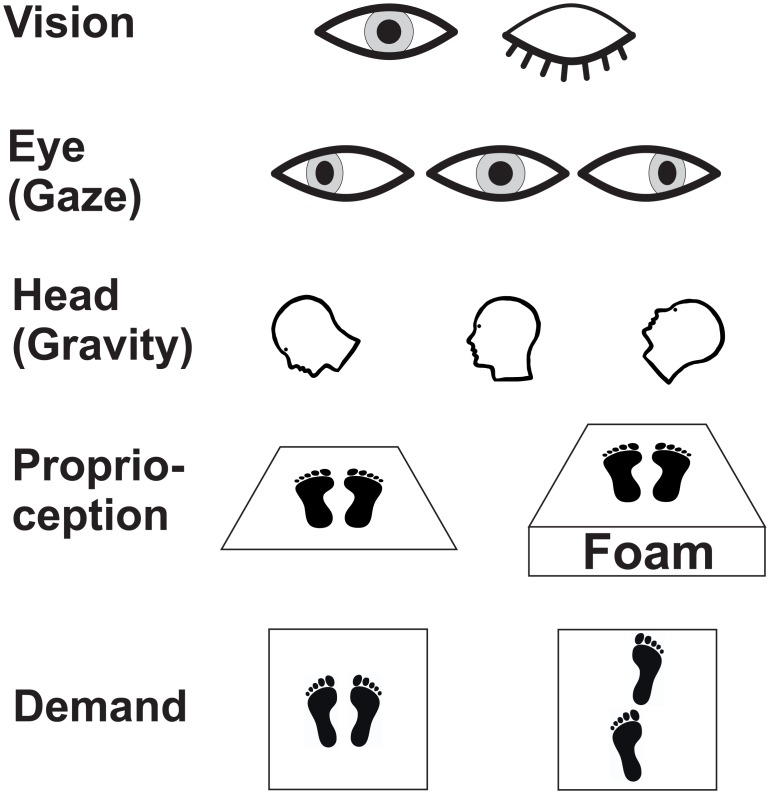
Study design. Experimental conditions are illustrated by symbols: visual input (eyes open/closed), gaze position (straight ahead, eccentric target positions), graviceptive (head down, erect, head up) and proprioceptive (platform vs. foam) input and, finally, different demands on postural control (parallel vs. tandem stance).

Posturography was recorded in the upright standing position with the hands hanging next to the trunk for 20 seconds under various experimental conditions (Fig 1) which differed in terms of (i) visual input (eyes open/closed; EO/EC), (ii) gaze position (left, gaze straight ahead, right, down and up midline; LEDs were illuminated in an aluminium cross of vertical and horizontal bars 60 cm in front of the participants’ forehead), (iii) graviceptive (head tilted up, down, head erect) and (iv) proprioceptive (platform vs. foam) input and (v) different demands on postural control (normal vs. tandem stance). Normal (parallel) stance referred to feet at an angle of about 30 degrees open to the stimulus wall and heals 3 cm apart (baseline stance condition).

Head position was adjusted to the following positions by an inclinometer [2]. This recording assured that the 3 different head positions (anteflection by 45°, upright head positions, 30° dorsoflection of the neck; with gaze straight ahead relative to head position) were maintained for the recording time. We used a slab of foam rubber (50 width, 60 cm length, height 10 cm, specific weight) for testing balance control under weakened proprioceptive feedback under two conditions: a) with the head erect, gaze fixation of LED at the gaze straight ahead position in the light and b) with the eyes closed.

### Posturography

We used a Kistler force platform (Model 9260AA6, Kistler Instrumente AG, Winterthur, Switzerland; 50 cm width, 60 cm length, height 10 cm) equipped with piezo-electric 3-component force sensors for recording postural changes during the above mentioned experimental conditions in a similar way as described elsewhere [16, 17]. Postural sway signals were bidirectionally filtered (50 Hz Gaussian filter) to eliminate low amplitude recording noise [18]. The platform recorded torques and sheer forces with six degrees of freedom using force transducers with an accuracy better than 0.5 N. The displacement of the center of pressure (CoP) in the medial-lateral (ML) and the anterior-posterior (AP) directions were recorded and the sum vector calculated using Matlab^®^. If not stated otherwise, results are given as the mean postural sway speed (PSS, in cm/s), calculated from the anterior-posterior (AP) and medio-lateral (ML) movements:

$$PSS=mean\left(\sqrt{{\left(AP_{i}-AP_{i-1}\right)}^{2}+{\left(ML_{i}-ML_{i-1}\right)}^{2}}*SamplingRate\right)$$

Postural sway was recorded in intervals of 20 s duration for off-line analysis (sampling frequency 250 Hz).

### Voxel based morphometry

The VBM data analysis was performed using the CAT (Computational Anatomy Toolbox for SPM; http://dbm.neuro.uni-jena.de/cat/) with SPM12 software package [19]. Pre-processing steps were applied as described before [20]. The spatial smoothing was set to 8 mm FWHM. The voxel size for the spatially normalized images was 1.5×1.5×1.5 mm^3^. Differences in regional grey matter volume (GMV) between healthy controls and patients were investigated applying voxel-wise two-sample t-tests. A statistical mask for the cerebellum was created using the automated anatomical labelling (AAL) atlas [21]. All modulated grey matter images were thresholded by 0.1. The cluster defining threshold was set at p = 0.001. The statistical analysis included N = 49483 voxels (voxel size 1.5×1.5×1.5mm^3^). A family-wise error correction (FWE) procedure was applied at the cluster level to correct for multiple comparisons (p<0.05 corrected). Age and gender were regressed out before the group and correlation analyses. Regression analyses were used to relate GMV changes to behavioural data (postural sway speed in EC and EO, slow phase velocity (SPV) of downbeat nystagmus, vertical and horizontal gain of smooth pursuit eye movements, SARA and disease duration). The statistical analysis of behavioural data was performed using Matlab^®^. We used cytoarchitectonic probability maps [22] and the SPM Anatomy toolbox [23] to specify anatomical locations.

### Statistical analysis

Statistical analyses were performed with SPSS (22.0.0.2; IBM Corp., Somer NY). Gaze (factor GAZE), head (factor HEAD) positions and proprioceptive input (factor PROPRIOCEPTION) were taken as within-subject factors, group as between-subjects factor and disease duration and clinical scores as covariates. In some comparisons sphericity requirement was violated. Therefore, we report F-values with Greenhouse-Geisser correction but report degrees of freedom (df) uncorrected in order to show the factorial analysis design. Statistical comparisons were performed parametric unless stated otherwise.

A multi-factorial ANOVA with the factors GAZE, HEAD, PROPRIOCEPTION, and TANDEM was performed. When the factors “Group” or “condition” were significant, the effect was characterized post-hoc by conducting ANOVAs on different pairs of factor levels of the factor. Significance levels of these tests were Bonferroni corrected for multiple testing. Statistical differences were regarded as significant for values p<0.05 if not stated otherwise. Error bars indicate standard error. Correlation analyses were performed using Spearman-Rho coefficient unless otherwise stated.

The effects of visual deprivation on postural stability were determined by *Romberg’s ratio* computing PSS with the eyes closed / eyes open [24].

## Results

Clinical scores are listed in S2 Table.

### Oculomotor behavior

Slow phase velocity (SPV) of DBN on gaze straight ahead was on average 2.25°/s ±0.46. It increased on lateral 4.72°/s ±0.73 and downward gaze 5.36 ±0.66. Smooth pursuit velocity gain was reduced during sinusoidal (0.2 Hz) horizontal [patients: 0.61±0.04; controls: 0.91±0.01; t(51) = 6.1; p<0.001] and vertical smooth pursuit eye movements [patients: 0.58±0.1; controls: 0.92±0.02; t(51) = 3.2; p = 0.003].

### Postural behaviour in different experimental conditions

ANOVA revealed a main effect of postural sway speed (PSS) for GROUP F(1,51) = 21.15; p<0.001), i.e. PSS was consistently larger in patients (4.449 ±0.244 cm/s) than in healthy control subjects (2.877 ±0.239 cm/s).

#### Target visibility

**Fig 2:**
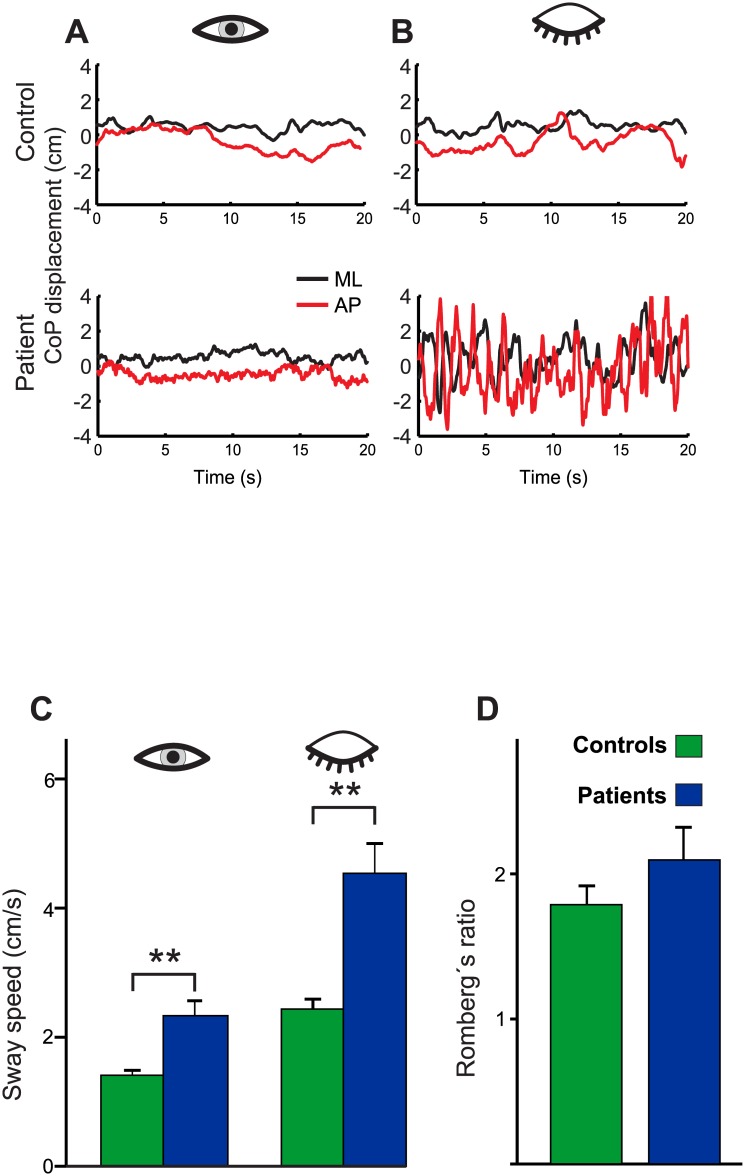
Postural sway as a function of target visibility. Original recordings of postural medio-lateral (ML, purple) and anterior-posterior (AP, green) center of displacement (CoP in mm) of a healthy control subject (upper trace) and a DBN patient (lower trace) on solid platform with eyes open (**A**) and closed (**B**). Group means ± standard error (SEM) are shown in (**C**) indicating significant larger PSS in patients but an indistinguishable increase of PSS in both groups on eye closure. Accordingly, Romberg’s ratio (**D**) is not different between groups.

There was a main effect for GROUP (F(1,51) = 22.73; p<0.001) and TARGET VISIBILITY (Eyes open/eyes closed; EO/EC) (F(1,51) = 48.22; p<0.001), i.e. PSS in patients and controls (solid platform, parallel feet, head upright) was significantly larger during eye closure than during eyes open. There was a significant interaction for TARGET VISIBILITY x GROUP (F(1,51) = 6.01; p = 0.018), i.e. PSS increased on eye closure more in patients than in controls (Fig 2A–2C). There was no significant difference for **Romberg’s ratio** (PSS ratio of EC/EO) between both groups (patients: 2.09±0.22; controls: 1.78 ±0.12; (T(51) = 1.177; p>0.2), i.e. the relative increase in PSS on eye closure is not different between the groups (Fig 2D).

#### Gaze dependency

**Fig 3:**
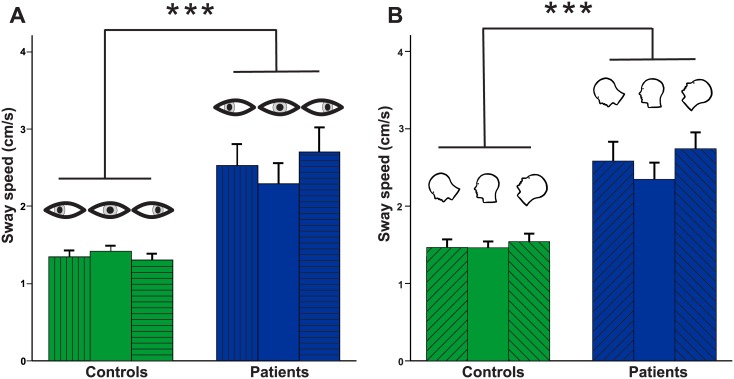
Postural sway as a function of horizontal gaze and head position (gravity). (**A**) Postural sway speed (PSS in cm/s) is shown for the gaze straight ahead position and right and left gaze positions (20°). PSS differed between groups but not within groups, i.e. PSS was not gaze dependent. (**B**) PSS is shown for different head positions in the straight ahead gaze position: head forward (45°), erect, and backward (30°) bended head position. PSS differed between groups but not within groups, i.e. PSS was not gravity dependent.

Patients showed significantly larger PSS (2.544±0.168 cm/s) than healthy controls (1.355±0.168 cm/s) (F(1,52) = 27.72; p<0.001) but there was no main effect for horizontal GAZE (F(2,51) = 1.14, p = 0.319). However, there was an interaction of horizontal GAZE x GROUP (F(2,51) = 3.62, p<0.036), i.e. PSS increased on lateral gaze in patients while it decreased in controls (p = 0.353)(Fig 3A). Post-hoc ANOVAs showed no main effect for GAZE within each group [patients: F(2,25) = 2.77, p<0.079; healthy control subjects: F(2,24) = 0.98, p = 0.36)]. There was neither a main effect for vertical GAZE (F(2,51) = 1,14, p>0.3) nor an interaction of vertical GAZE x GROUP (F(2,51) = 0.53, p>0.5).

#### Head position (gravity)

Apart from the main effect for GROUP [eyes open: F(1, 51) = 26.16, p < 0.001: eyes closed: (F(1, 51) = 19.74, p < 0.001)] there was no main effect of HEAD POSITION (gravity) on PSS. As in the head erect condition, there was a main effect for target visibility (EO/EC) (F(1,51) = 61.81, p < 0.001) and an interaction EO/EC x GROUP (F(1,51) = 7.23, p = 0.01), i.e. patients show larger PSS on EC as compared to EO in relation to controls (F(1,51) = 25.92, p < 0.001). There was no interaction of HEAD POSITION x TARGET VISIBILITY or HEAD POSITION x TARGET VISIBILITY x GROUP (p always > 0.4) (Fig 3B).

#### Proprioceptive deprivation (foam)

Nine patients required assistance during proprioceptive deprivation and tandem stance to prevent falls. Accordingly, they were excluded from the analysis. In the remaining patients (n = 18), apart from the main effect for GROUP (F(1, 42) = 21.74, p < 0.001), there was a main effect of Proprioceptive deprivation (foam, F(1,42) = 145.82, p < 0.001), a main effect for TARGET VISIBILITY (F(1,42) = 59.11, p < 0.001) and an interaction of TARGET VISIBILITY x GROUP (F(1,42) = 18.17, p < 0.001). There was no triple interaction for TARGET VISIBILITY x GROUP x Proprioceptive deprivation (F(2,43) = 0.004; p>0.9).

**Fig 4:**
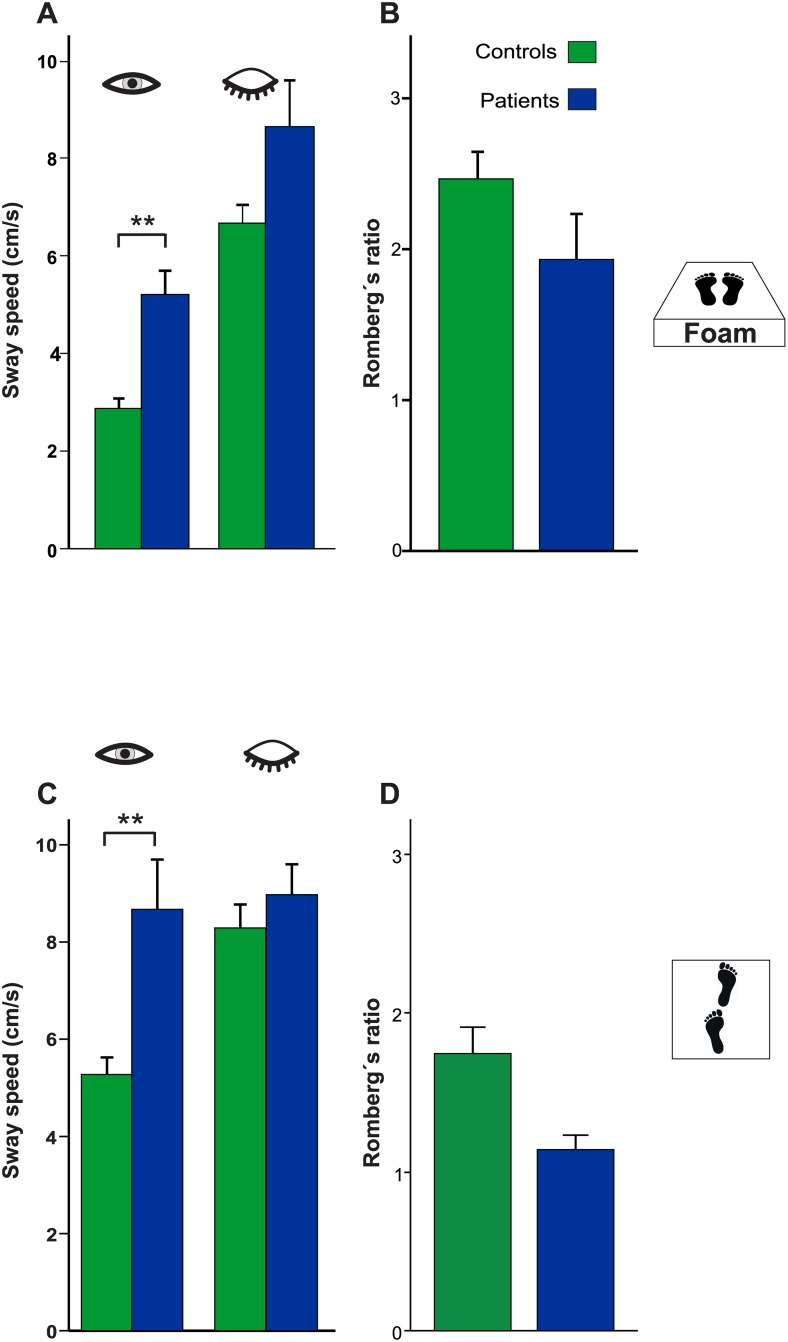
Postural sway as a function of proprioceptive deprivation and higher demand (tandem stance). PSS increased with attenuated proprioceptive input (standing on foam) in both groups (**A, B**). PSS of patients was significantly higher in both conditions (eyes open and closed). However, there was no significant difference in the increase in PSS between both groups in the foam condition; both with the eyes open and closed (Romberg’s ratio). (**C**) PSS of patients was significantly higher during tandem stance in both conditions (eyes open and closed) but Romberg’s ratio did not differ between groups (**D**).

With the eyes open, there was a main effect for Proprioceptive deprivation (foam) (F(1,42) = 185.88; p<0.001); i.e. patients showed significantly larger PSS on foam (5.289±2.046 cm/s) than healthy controls (2.934±1.028 cm/s) (Fig 4A). There was a significant interaction GROUP x Proprioceptive deprivation (F(1,42) = 20.62, p<0.001). With the eyes closed, there was a main effect for GROUP (F(1,42) = 11.51, p = 0.002) and Proprioceptive deprivation (foam) (F(1,42) = 77.11, p<0.001) but no interaction (p>0.7). However, Romberg’s ratio on foam did not differ (patients: 1.93±0.3; controls: 2.52±0.18; T(42) = -1.78, p = 0.081).

#### Tandem stance

There was a main effect of TANDEM STANCE (F(1, 42) = 201.25, p < 0.001) and for TARGET VISIBILITY (F(1, 42) = 35.98, p < 0.001) as well as an interaction for GROUP x TANDEM STANCE (F(1,42) = 17.426, p< 0.001).

With the eyes open, there was a main effect for GROUP (F(1, 42) = 16.804, p < 0.001) and TANDEM (F(1,42) = 118,77, p<0.001) and an interaction of GROUP x TANDEM (F(1, 42) = 7.00, p = 0.011), i.e. patients showed significantly larger PSS on tandem stance (8.672 ± 4.354 cm/s) than healthy controls (5.332±1.835 cm/s) (Fig 4C and 4D) (T(42) = 2.78, p = 0.008). There was no significant difference for Romberg’s ratio (PSS ratio of EC/EO) in tandem stance between both groups (patients: 1.14±0.09; controls: 1.74 ±0.17; (T(51) = 1.17, p>0.2). The ratio between PSS during TANDEM vs. normal standing did not differ (patients: 4.341 ± 0.65; controls: 3.809±0.24; T(42) = 0.868, p>0.39). With the eyes closed, there was a main effect for GROUP (F(1, 42) = 7.02, p = 0.011) and TANDEM (F(2,42) = 151,20, p<0.001) and an interaction of TANDEM x GROUP (F(1,42) = 4.86, p = 0.033). The ratio of PSS during TANDEM vs. normal standing with the eyes closed differed (patients: 2.412 ± 0.31; controls: 3.841 ± 0.37; T(42) = 2.76, p = 0.008) but not with the eyes open.

#### Clinical Scores

PSS increased with DBN (slow phase velocity) with the eyes closed (r = 0.587; p = 0.001) (S1A Fig) but not with the eyes open (r = 0.033; p = 0.871) (S1B Fig). PSS (parallel feet, solid platform, head erect) increased with the severity of clinical ataxia (correlation with SARA Score; r = 0.621, p = 0.001) (S1C Fig) and the Tinetti Score (r = -0.528, p = 0.005). Postural sway was independent of age, vestibular-induced disability (VHQ, VSS) and cognitive scores (MOCA; p always > 0.3, r always < 0.26). It did not correlate with disease duration (r = 0.331, p = 0.142).

#### Voxel based morphometry

**Fig 5:**
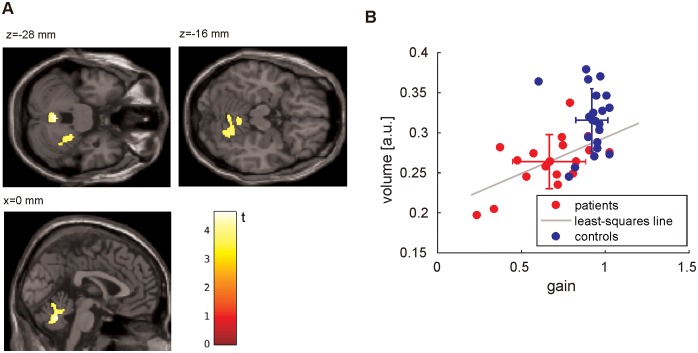
Relation of behavioral parameters to cerebellar grey matter volume changes. Significant (FWE-corrected) gray matter volume reductions in DBN patients (healthy controls > patients) are depicted in cerebellar vermis (lobules VI and VIII and deep cerebellar nuclei) and cerebellar hemispheric lobules (V-VI) in axial and sagittal slices (p<0.001). GMV reduction (ROI of vermal cluster) increases with stronger impairment (gain) of smooth pursuit eye movements (B). Blue (healthy control) and red (patients) circles show individual data, crosses indicate the mean of gain and ROI-based volume ± standard deviation.

There was significant GMV reduction in cerebellar vermis and cerebellar hemispheric lobules V-VI (Fig 5) (S3 Table). According to cytoarchitectonic probability maps [23], the cerebellar cluster (1862 voxel) covered primarily the cerebellar lobules V-VII (right VI: 27.3%, right V: 16.8%; left VIIb 7.1%) and vermis [right 10.5% and left (8%) lobule VIIIa]. The maximum of GMV reduction was found in the vermis (2–64–24) in lobules VI (41%) and VIIIa (16%) and deep cerebellar nuclei, i.e. fastigial (13%) and interpositus nucleus (18%). Using the cluster defined region of interest there was a significant decrease of GMV with increasing horizontal smooth pursuit eye movement impairment (rho = 0.58; p = 0.0145; Fig 5B). There was no significant correlation between GMV change, postural sway speed, slow phase velocity of downbeat nystagmus, vertical smooth pursuit eye movement gain, SARA and disease duration (p>0.05).

## Discussion

Balance control requires an integration of multiple sensory signals and their interaction with motor control of stance[25]. Sensory control of stable body posture is maintained by error signals deriving from the vestibular, visual and proprioceptive system. They need to be processed, integrated and weighted as a function of individual demand which may change in disease.

As a main result, patients consistently showed increased postural sway throughout all experimental conditions as compared with healthy controls. Postural ataxia was independent of graviceptive or gaze contingency. Data do not support hypothesis #1 stating that postural unsteadiness in DBN is related to the severity of visual impairment by oscillopsia since postural sway did not increase with the eyes open or on lateral gaze. In line with *Hypothesis #2*, postural sway was consistently larger with visual deprivation on eye closure. It increased with the severity of oculomotor impairment (DBN) on eye closure but not during fixation in the light.

### Visual control of posture

#### Gaze contingency

In healthy subjects, eccentric eye positions elicit deviation from the intended path[5] but this has not been systematically investigated during stance. The gaze contingency of DBN has been thought to account for patients’ blurred vision and oscillopsia [4]. The retinal slip signal is misinterpreted as motion of the visual scene since involuntary ocular oscillations are not associated with an efference copy signal. Accordingly, visual motion could destabilize postural control.

In contrast to this hypothesis, we did not find a significant change of postural sway speed on lateral and downward gaze. There was only a weak interaction group x gaze which is related to a trend of oppositely directed changes of gaze on PSS in patients and controls. This interaction was not found in vertical gaze. Since PSS was not related to the DBN amplitude (SPV) during fixation of space-stationary targets but strongly correlated with SPV on eye closure, postural ataxia in DBN is probably independent of retinal error signals by oscillopsia. Alternatively, postural sway in DBN could be related to the static eye position [25], specifically to the efference copy of the eye movement signal irrespective of retinal slip [8, 26]. However, this explanation is not supported by our patients since postural ataxia did not significantly change with eccentric eye positions.

#### Visual cues

Under normal conditions, postural stability is better with visual fixation of space-fixed targets than in darkness [27] implying that visual information can be used to reduce and optimize postural stability [28]. The severe increase of postural ataxia on eye closure may be either attributed to visual deprivation or an increase of DBN in darkness [29]. However, the indistinguishable increase in postural unsteadiness of patients and healthy controls (without nystagmus) make the latter explanation unlikely.

The Romberg ratio (EC/EO) is used as an indicator of visual and proprioceptive contribution to postural stability [17]. Romberg’s ratio did not differ between our DBN patients and controls. Despite DBN-related oscillopsia, patients can use visual signals to stabilize posture in the same way as healthy persons do. This is in line with related studies showing that gait impairments of DBN patients deteriorate during absent visual control [7]. In line with our current posturographic data, DBN patients even utilize visual information better than healthy controls. As a consequence, oscillopsia during walking cannot solely account for the gait disorder of DBN patients. This may be explained by a shift in weighing of visual information as part of a compensatory strategy in DBN patients. From a clinical point of view, the strong visual dependency of postural control resembles proprioceptive ataxia of stance in patients with polyneuropathy rather than patients with cerebellar ataxia. This in line with ataxia of gait in DBN patients which clearly differ from cerebellar patients with additional limb involvement [7].

### Vestibulo-spinal control of posture

One reason for DBN is a tone imbalance of central (flocculus) vestibular pathways of vertical eye movements [3]. This tone imbalance encompasses utricular signals [1, 30]. Physiologically, head tilts in the pitch plane increase postural sway in the light and darkness [31]. The vestibulo-cerebellum minimizes an overacting otolith-ocular reflex elicited by head positions in the pitch plane and cancels an inherent upward ocular drift that is independent of gravity-modulated otolith signals [3]. In DBN, the cerebellar influence on utriculus-ocular reflex is disinhibited. Specifically, DBN consists of a spontaneous vertical eye velocity drift and a gaze- and gravity-dependent component. The gravity-dependent component of DBN is modulated as a function of body [32] position along the pitch plane. Potassium channel blockers, 3,4-diaminopyridine and 4-aminopyridine, decrease DBN in gravity-dependent head positions by reducing the overactive otolith-ocular reflex and concomitant reduction of oscillopsia [2, 33]. They also improve postural balance [34, 35]. Unlike expected we did not find changes in postural stability with different head positions. Therefore, increased PSS cannot be solely attributed to a disinhibited otolithic-spinal mechanism of postural control.

### Proprioceptive control of posture

Weakening somatosensory feedback by standing on foam increases postural instability. Postural sway on foam increased in both groups considerably. The larger increase of postural sway in healthy controls reflects a ceiling effect since proprioceptive deprivation already heavily destabilized patients during fixation in the light. Despite the pronounced utilization of visual information to stance it cannot compensate for deficient proprioceptive input. Interestingly, Romberg’s ratio did not differ between the groups. This implies that patients can use proprioceptive signals as controls do, irrespective of the visual condition. This distinguishes them from patients with peripheral vestibular disease, e.g. in Meniere’s disease [36], or proprioceptive postural imbalance, e.g. polyneuropathy [37]. Thus, DBN patients can partially use visuo-proprioceptive integration probably by reweighting sensory inputs but not sufficiently to stabilize stance [38].

### Increased motor demand on postural balance (tandem stance)

Postural sway increased with demand and motor task complexity, i.e. tandem stance with the eyes open and closed. Given that DBN patients already showed severe postural sway in the light (EO) the stronger increase of PSS in healthy controls during tandem stance with the eyes closed probably reflects a ceiling effect again. Noteworthy, it is the motor task complexity which destabilized DBN patients most. Nine patients had to be removed from analysis because of required external stabilization. This might suggest that postural instability is largely caused by inappropriate motor signal commands.

### Combined tasks in postural control

If patients and healthy controls do not show differences with deprivation of single sensory components, one might speculate that postural ataxia in DBN is a failure of multisensory integration of proprioceptive, visual and vestibular signals. This is supported by the exclusion of several patients whose balance control was destabilized by combined visual and proprioceptive. Patients with cerebellar disease show worsening of their postural control on a solid platform in dual tasks conditions, i.e. by employing an additional working memory task [39]. This is of clinical importance as the higher postural sway during dual tasks increase the risk of falls [39]. Idiopathic DBN is often acquired in the elderly who spend increasing amounts of their cognitive resources into the coordination of balance [40]. In cerebellar disease, this resource allocation might be even more difficult due to inherent postural ataxia. Finally, the dynamics (e.g. delay) of sensory re-weighting is crucial in human postural control, both in health and disease [38, 41]. Thus, impaired motor control and re-weighting of multisensory signals might account for postural ataxia in DBN.

### Structural morphological changes

For the first time we show a significant (FWE-corrected) GMV reduction in cerebellar vermis (lobules VI/VIII) and hemispheric lobules V-VI in patients with idiopathic downbeat nystagmus. This is in line with a preliminary report of 11 DBN patients which showed a trend (uncorrected data p<0.01) for vermal atrophy (lobules V,VI) [42]. Cerebellar smooth pursuit eye movement neurons are located in the oculomotor vermis (lobules VI-VII) and deep cerebellar nuclei [43]. Accordingly smooth pursuit gain significantly decreased in our patients with increasing vermal / deep cerebellar nuclei atrophy. DBN has been reported with lesions of the flocculus [2, 3] but not in this mid-vermal region. Accordingly, nystagmus slow phase velocity of our patients did not correlate with vermal atrophy.

Neither hemispheric nor vermal atrophy was linked to the considerable postural ataxia in our patients, irrespective of the visual condition.

## Conclusions

In conclusion, impaired postural control in our DBN patients is neither related to modulations of single components characterizing DBN (gaze- or gravity dependence) nor to deprivation of single sensory (visual, proprioceptive) inputs usually stabilizing stance. Abnormal integration or re-weighting of multisensory signals and/or inappropriate cerebellar motor commands might account for postural ataxia in DBN. From this perspective, physical therapy should engage multisensory rather than uni-sensory stimulation in neurorehabilitation.

## Supporting Information

S1 Fig Postural sway speed as a function of downbeat nystagmus and clinical scores of cerebellar impairment. With the eyes open (**A**) postural sway speed was not related to nystagmus (slow phase velocity °/sec). In contrast, slow phase velocity (SPV) increased during eye closure (**B**) and with the severity of clinical ataxia (SARA-Score) (**C**).
(TIF)

S1 Table Clinical data of all DBN patients. Modulation of DBN by graviceptive input (head pitched forward, backward) is indicated by mild (+) or strong (++) increase of SPV of DBN in the head bended forward position or its absence (-). Clinical examination particularly looked for extracerebellar signs, e.g. polyneuropathy or peripheral vestibular lesions. Assessment of clinical disability by cerebellar dysfunction are given by scale for the assessment and rating of ataxia (SARA), the Tinetti balance and gait evaluation score, the Vertigo Symptom Scale (VSS) and Vertigo Handicap Questionnaire (VHQ in %).
(XLS)

S2 Table Mean values (±SE) for disability and handicap scores. Scale for the assessment and rating of ataxia (SARA); Vertigo Symptom Scale (VSS), vertigo handicap questionnaire (VHQ), Tinetti Assessment Test[10, 11], Montreal Cognitive Assessment (MOCA).
(XLSX)

S3 Table Local grey matter volume in healthy controls compared to DBN patients (HC>DBN). (DOCX)
