# Supplementary material for: Postural Ataxia in Cerebellar Downbeat Nystagmus: Its Relation to Visual, Proprioceptive and Vestibular Signals and Cerebellar Atrophy
Source: PLoS One. 2017 Jan 5;12(1):e0168808. doi: 10.1371/journal.pone.0168808 (PMC5215796; doi:10.1371/journal.pone.0168808)
Supplement: S3 Table — (DOCX) [file pone.0168808.s004.docx]

**Supplementary Table e-3**

Local grey matter volume in healthy controls compared to patients (HC>DBN).

cluster-level peak-level

p(FWE-corr) *K*_E_ p(uncorr) p(FWE-corr) T p(uncorr) x y z (mm)

0.001 1862 0.001 0.035 4.66 0.000 2 -60 -28

0.109 4.17 0.000 6 -68 -34

0.128 4.10 0.000 22 -66 -14

The table shows a maximum of 3 local maxima more than 8.0 mm apart.
